# The Association Between Antipsychotics and Weight Gain and the Potential Role of Metformin Concomitant Use: A Retrospective Cohort Study

**DOI:** 10.3389/fpsyt.2022.914165

**Published:** 2022-05-24

**Authors:** Alqassem Y. Hakami, Razaz Felemban, Rami Ghazi Ahmad, Abdulrahman H. Al-Samadani, Hassan K. Salamatullah, Jamil M. Baljoon, Loay J. Alghamdi, Mostafa H. Ramadani Sindi, Mohamed Eldigire Ahmed

**Affiliations:** ^1^College of Medicine, King Saud Bin Abdulaziz University for Health Sciences, Jeddah, Saudi Arabia; ^2^King Abdullah International Medical Research Center, Jeddah, Saudi Arabia; ^3^Psychiatry Section, Department of Medicine, Ministry of National Guard-Health Affairs, Jeddah, Saudi Arabia; ^4^College of Science and Health Professions, King Saud Bin Abdulaziz University for Health Sciences, Jeddah, Saudi Arabia

**Keywords:** antipsychotics, weight gain, weight loss, metformin, obesity

## Abstract

**Background:**

Obesity and its complications are associated with several adverse effects that may cause a serious impact on health. Antipsychotics-induced weight gain (AIWG) is one of the major, yet often neglected side effects of first and second generations antipsychotics. Importantly, several researches have shown metformin to be effective in managing weight gain especially, with AIWG. This study investigated the effect of antipsychotics use on weight gain and the theory of metformin concomitant use on the prevention of AIWG.

**Methods:**

A retrospective cohort review of the medical records of patients from the psychiatry outpatient clinics in the King Abdulaziz Medical city, a tertiary hospital in Jeddah from May 2016 to August 2021. The population of patients in Psychiatry section was 4,141. The sampling technique was a non-random consecutive sampling technique. Moreover, the included patients’ records were divided to group 1 (patients on antipsychotics) and group 2 (patients using antipsychotics with Metformin).

**Results:**

According to the study criteria, 395 patients’ records were included. A total of 309 (78%) patients were using antipsychotics without metformin, which in this study were depicted as group 1. In addition, a total of 86 (22%) were using antipsychotics with metformin, which in this study were assigned as group 2. Out of Group 1 patients (*n* = 309), only 67 patients experienced weight loss (21.68%), 43 remained with no weight change (13.92%), and 199 experienced weight gain (64.4%). Out of Group 2 patients (*n* = 86), 35 patients experienced weight loss (40.7%), 18 patients remained with no weight change (20.93%), and 33 experienced weight gain (38.37%). In addition, group 1 had a mean weight change of 2.5 kg, whereas group 2 had a mean weight change of −0.04 kg.

**Conclusion:**

Statistical analysis revealed that patients on antipsychotics alone experienced weight gain, whereas the concomitant use of metformin showed reduction in the weight gain tendency. Thus, study outcomes indicate that concomitant use of metformin with antipsychotics might significantly reduce the AIWG.

## Introduction

In the past few decades, the prevalence of obesity has significantly increased in Saudi Arabia (1). Furthermore, obesity leads to the detrimental effects of metabolic syndrome such as cardiovascular diseases and type II diabetes mellitus (DM) (2). There are many factors that contribute to obesity, including the use of some antipsychotic drugs such as *olanzapine* and *clozapine* (3).

Antipsychotics, also known as neuroleptics, are majorly used to treat psychosis and mainly schizophrenia (3). The mechanism of antipsychotics-induced weight gain (AIWG) is generally hypothesized by the alteration of glucose metabolism and increasing cholesterol and triglyceride levels. Thus, increase the chance of insulin resistance and may cause arterial hypertension disposing to metabolic syndrome (4–6). Specifically, antipsychotics affect neuropeptides linked with appetite control and energy metabolism such as leptin, adiponectin, and ghrelin (3, 5). Changes in these neuropeptides’ levels have shown a direct impact on weight gain hence increasing the release of triglycerides and Very Low-Density Lipoprotein (VLDL) (5, 6).

First generation (typical) antipsychotics mainly act upon the antagonism D2 receptor and serotonin (5HT) to a lesser extent which commonly lead to extra-pyramidal symptoms and tardive dyskinesia as adverse effects of these drugs. Second generation (atypical) antipsychotics mainly block serotonin (5-HT) and norepinephrine (α1 and α2). Atypical antipsychotics also show a reduction in extra-pyramidal symptoms compared to typical antipsychotics because of lower affinity to D2 receptor antagonism thus portraying more metabolic rather than neurologic side effects (7). One of the causes of Antipsychotics-induced weight gain (AIWG) is increased food intake (8). Besides antipsychotics, there are other lifestyle factors that may be attributed to the weight gain in psychiatric patients. For example, paranoia or hospitalization of patients with schizophrenia may force them to be isolated in a sedentary lifestyle (9).

Evidence have shown that the rate of weight gain is high during the first 6 months of antipsychotics treatment establishment and progress throughout the treatment period (10). Also, reports have determined that *clozapine* and *olanzapine* are associated with a high risk of weight gain, whereas the antipsychotics associated with a low risk of weight gain are *aripiprazole*, *lurasidone*, and *ziprasidone* (11, 12). The role of antidiabetic drugs in the management of AIWG has been introduced in several studies. *Metformin* improves the action of insulin in the liver which leads to decreased hepatic glucose production, increases peripheral utilization, and decreases appetite (13, 14). Moreover, *Metformin* reported that it can decrease antipsychotic-induced weight gain by decreasing insulin resistance and appetite (15). Previous report demonstrated that using *metformin* reduced body weight, body mass index (BMI), and insulin resistance index (IRI) (15). The insulin resistance can increase with continuous weight gain and with the chronic use of antipsychotics medications. Despite the notion that using antidiabetic drugs is suggested to decrease the weight gain following antipsychotic use, some antipsychotic medications have been linked to a more favorable weight effect. For example, in schizophrenic patients, the use of alternative medications such as *aripiprazole* can have a low risk of increasing weight (16, 17).

The prevalence rate of DM is alarmingly increasing worldwide (18). Moreover, one-fourth of the adult population of Saudi Arabia is affected by DM. This number is predicted to rise to more than double by the year 2030 (19). Moreover, the effect of antipsychotics on weight gain is not well-studied in Saudi Arabia. In addition, the literature review revealed that only few studies in the region have investigated the effect of metformin concomitant use on antipsychotic induced weight gain (20). This study aims to investigate the effect of antipsychotics on the weight gain, and the effect of Metformin in counteracting AIWG in patients from National Guard Health Affairs (NGHA) in Jeddah, Saudi Arabia.

## Materials and Methods

### Ethical Approval

This study was approved by King Abdullah International Medical Research Center (KAIMRC) institutional review board (study number: SP21J/112/03).

### Design and Setting

**FIGURE 1:**
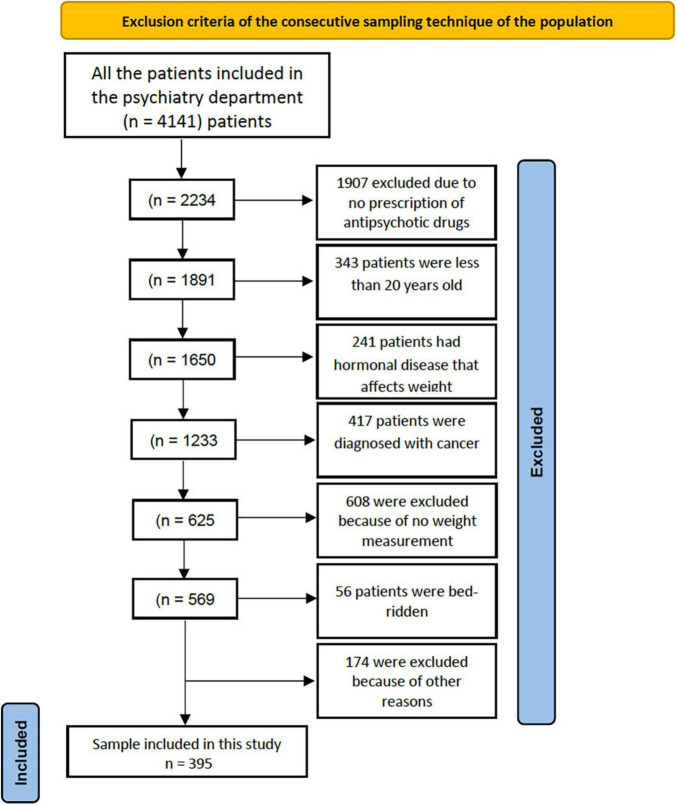
Illustrates the entire population on which inclusion and exclusion criteria were applied. The population included 4,141 patients. The authors went through each individual patient in BestCare system to match the criteria and only 395 patients out of the population met the criteria.

This is a retrospective cohort study that examined the medical records of patients who received antipsychotic medications in the psychiatric section in National Guard Health Affairs (NGHA), a tertiary hospital, in King Abdulaziz Medical City in Jeddah (KAMC-J), from May 2016 to August 2021. The sampling technique was a non-probability consecutive sampling technique and the patients were divided into two groups. The first group included patients on antipsychotic medications only, and the second group was patients taking antipsychotics with metformin. Then using the hospital information system (BestCare), patients’ data were collected according to the variables in the data collection sheet. These variables include, gender, age, weight measurements, weight recording interval, and the name of antipsychotic medications were recorded for each patient. Following the data collection phase, data were initially encoded within an excel sheet to identify any missing data. The initial number of patients examined in the study was 4,141 patients. The inclusion criteria consist of patients using antipsychotics (group 1) and patients using antipsychotics with metformin (group 2). The exclusion criteria consist of physically disabled patients, hormonal disorders causing weight fluctuations such as thyroid disorders, patients below the age of 20, loss of follow up after prescribing antipsychotics, patients diagnosed with any type of cancer, any patients using antipsychotics without a weight record, pregnancy, and patients with a history of bariatric surgery. The total sample size after exclusion criteria is shown in Figure 1. The sample size was calculated by Openepi website version 3 using the confidence interval of 95%, and a power of study 80%. According to de Silva et al. (21) and de silva et al. (15), the first group who used antipsychotics alone had a mean of –1.56 kg with a standard deviation of 4.29 (15, 21). On the other hand, the second group who used antipsychotics with metformin had a mean weight gain of 1 kg, and the standard deviation was 2.69. Since the ratio of group 2/group 1 is 1.06, the required minimum sample size is 30 and 32, or groups 1 and 2, respectively. However, since this study is a consecutive study, all the patients who met the inclusion criteria and did not meet the exclusion criteria were involved in the study with numbers of 309 and 86 for group 1 and group 2, respectively.

### Data Analysis

The applications used in this study are J Macintosh Project (JMP) pro 15.2.0 and IBM Statistical Package for the Social Sciences (SPSS) statistics 28.0.1.1 (14). Regarding the descriptive categorical data, which are gender, age, weight interval, diagnosis, name of the antipsychotic drug, and metformin usage, were depicted by using frequency and percentages. The dependent factor of this study is the weight difference between “before using antipsychotics” and “after using antipsychotics” in both group 1 and group 2. The weight difference was depicted numerically by the mean and standard deviation and categorically by three categories which are “increased weight, “no difference in weight,” and “decreased weight.” For the univariable inferential statistics, parametric data were depicted by chi-square, *T*-test, and one-way ANOVA. Non-parametric inferential statistics are depicted by Mann–Whitney U test, Wilcoxon signed-rank test, and Kruskal–Wallis test. Regarding multivariable inferential statistics, binary logistic regression model was used for comparing different variables. *P*-values less than 0.05 were considered statistically significant.

## Results

A total of 395 patients who visited the psychiatry clinic in NGHA during the period of May 2016 to August 2021 met the inclusion criteria of the study. Regarding the weight interval, patients were divided into four groups according to the weight measurement, which are 1–3 months, 4–6 months, 7–9 months, and 10–12 months. The most common weight intervals were in both group 3–6 months and 9–12 months with a number of 111 patients (23%) each. In addition, a total of 105 patients (27%) were between 30 and 39 years age category. Moreover, around 62% of the patients included in the study were males. Regarding the medications reported in the study, 118 (30%) of the patients used quetiapine as one of the main options in the management plan for their psychiatric condition. Importantly, 104 (27%) of the sample in the study were reported as patients with depressive disorders. Patients’ demographic characteristics are illustrated in Table 1.

**TABLE 1:** Patient demographic characteristics, medical conditions, and antipsychotics.

Parameter	Values
**Age (*n* = 395)**	
20–29	73 (18%)
30–39	105 (27%)
40–49	97 (25%)
50–59	62 (16%)
60–69	34 (9%)
>70	24 (6%)
**Gender (*n* = 395)**	
Male	244 (62%)
Female	151 (38%)
**Group (*n* = 395)**	
Group 1 (antipsychotic)	309 (78%)
Group 2 (antipsychotic with metformin)	86 (22%)
**Weight recording interval (*n* = 395)**	
Low (0–3 months)	89 (23%)
Middle (3–6 months)	111 (28%)
High (6–9 months)	84 (21%)
Very high (9–12 months)	111 (28%)
**Medical psychiatric condition (*n* = 392)**	
Depressive disorders*	104 (27%)
Bipolar disorder	54 (14%)
Psychotic disorders**	54 (14%)
Prescribed for non-psychiatric disease	30 (8%)
Anxiety disorders***	29 (7%)
Mixed anxiety and depression	9 (2%)
Obsessive-compulsive disorder	7 (2%)
Substance misuse	4 (1%)
Post-traumatic stress disorder	3 (1%)
Autism	3 (1%)
Personality disorder	2 (1%)
Somatic disorder	2 (1%)
Pseudodementia	1 (0%)
Insomnia	1 (0%)
Attention deficit hyperactivity disorder	1 (0%)
Multiconditions of two or more of the above diagnosis	88 (22%)
**Antipsychotics (*n* = 395)**	
Quetiapine	118 (30%)
Olanzapine	87 (22%)
Risperidone	42 (11%)
Aripiprazole	37 (9%)
Sulpiride	30 (8%)
Trifluoperazin	15 (4%)
Haloperidol	5 (1%)
Multidrug combination of two or more of the above medications	61 (15%)

*Depressive Disorders: Major Depressive Disorder and its subtypes (MDD), Depressive neurosis, Postpartum Depression.

**Psychotic Disorders: Schizophrenia, Schizoaffective Disorder.

***Anxiety Disorders: Generalized Anxiety Disorder (GAD), Agoraphobia, Claustrophobia, Panic Disorder, Social Phobia.

**FIGURE 2:**
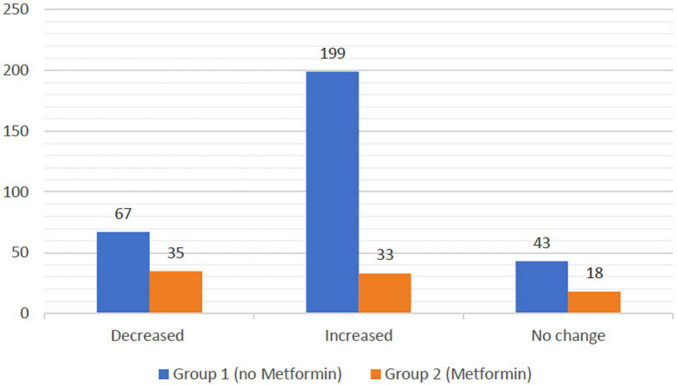
Represents the weight difference among group 1 and group 2 patients categorically. Group 1 revealed increase in the weight with 199 participants, whereas group 2 depicted much less weight gain with 33 participants only. In addition, 67 participants from group 1 have shown weight reduction in contrast to 35 participants from group 2. Importantly, only 43 and 18 participants from both group 1 and group 2 revealed no weight change during the study.

The statistical analysis revealed that group 1 patients had a mean weight change of +2.5 kg (95% CI = 1.94–3.06), whereas group 2 patients had a mean weight change of −0.04 kg (95% CI = −1.09 to 1.02) as illustrated in Table 2. Out of 309 patients in group 1, 67 (21.68%) patients experienced weight loss, 43 (13.92%), remained with no weight change, and 199 (64.4%) experienced weight gain. Out of 86 patients of group 2, 35 (40.7%) patients experienced weight loss, 18 (20.93%) patients remained with no weight change, and 33 (38.37%) experienced weight gain as illustrated in Figure 2. Importantly, statistical analysis showed no significant association between metformin usage and change of weight interval.

**TABLE 2:** Comparison between mean of weight change between antipsychotics alone vs. antipsychotics with metformin.

Variable	Antipsychotics use alone (*n* = 310)	Antipsychotics use with metformin (*n* = 89)	Significance
Mean	2.5 kg	−0.04 kg	*P* < 0.0001
Standard deviation (SD)	0.28	0.54	
95% CI	1.94–3.06 kg	−1.09 to 1.02 kg	

**FIGURE 3:**
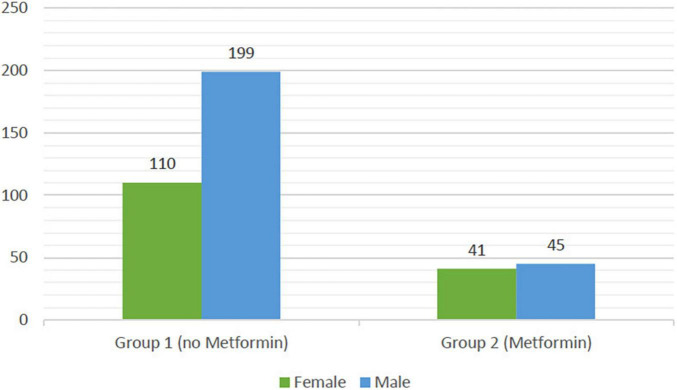
Illustrates the difference between male and female participants in metformin concomitant use. The 199 male participants formed the majority of group 1 vs. the 110 female participants. In group 2 the number of participants showed some similarities with 45 male and 41 female participants.

There is no significant association between gender and weight difference either in quantitative data or qualitative univariable analysis. However, a significant difference was reported between gender and metformin usage as illustrated in Figure 3. Thus, statistical analysis revealed that female patients on metformin with antipsychotics counted as 41 out of 154 (27.15%), whereas only 45 out of 244 (18.44%) of male patients had a metformin concomitant used with antipsychotics.

**FIGURE 4:**
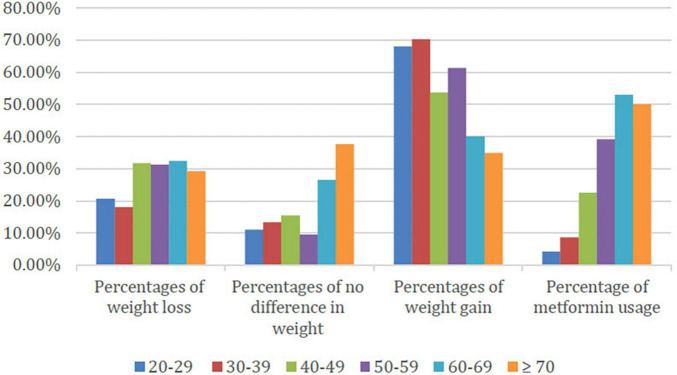
Demonstrates the changes in body weight and metformin use in different age groups of the participants. Weight loss was more prominent in the age groups of 40 and above (approximately 30% reduction). No weight changes were most common among the two oldest age groups (60–69 and ≥70) with frequencies of 26.47 and 37.50%, respectively. The youngest two age groups (20–29 and 30–39) showed the highest frequency of weight gain of 68.06 and 70.30%, respectively. The percentage of concomitant metformin use increased throughout the age groups with the youngest having the least frequency and the two oldest age groups (60–69 and ≥70) reported the highest frequencies of 52.94 and 50%, respectively.

Regarding the age variable, a total of 50 out of 73 (68.49%) patients in the 20–29 years age group had an average weight increase of +4.66 kg (95% CI 3.54–5.78). In contrast, the least affected group with weight gain were geriatric patients aged 60–69 with 11 patients out of 34 (32.35%) with decreased weight, and the mean of weight difference in this group is +0.15 kg (95% CI −1.5 to 1.79) as illustrated in Table 3. Moreover, the study outcomes revealed that metformin was prescribed more in elderly population as compared to other age groups. Of note, 18 out of 34 (52.94%) and 12 out of 24 (50%) of patients were reported to use metformin with antipsychotics in the following age groups, 60–69 years and more than 70 years, respectively. However, only 3 patients out of 73 (4.11%) in the 20–29 group were reported to use metformin with antipsychotics, the data illustrated in Figure 4. Importantly, statistical analysis revealed no significant association between age and weight interval. There was no significant association between group 1 patients and weight interval in qualitative nor quantitative analysis.

**TABLE 3:** Changes in the Participants’ body weight.

Age group	Mean weight difference (kg)	95% CI	Standard deviation (SD)	Significance
20–29	4.66	3.54–5.78	0.57	*P* < 0.0001
30–39	2.59	1.66–3.51	0.48	
40–49	0.95	−0.01 to 1.92	0.50	
50–59	0.78	−0.41 to 1.98	0.62	
60–69	0.15	−1.49 to 1.78	0.84	
≥70	0.35	−1.59 to 2.31	0.99	

In the binary logistic regression model, the independent variables were the usage of metformin, gender, age, and weight interval. The two dependent variables were decrease/no change in weight, and the other variable was increase in weight. Patients who used antipsychotics alone had an odds ratio of 2.35 to develop weight gain in comparison to patients who used antipsychotics with metformin. In addition, 20–29-year-old patients had a 3.46 odds ratio to develop weight gain in comparison to patients who aged ≥70 years. Similarly, patients in the 30–39-year-old age group had a 3.58 odds ratio to develop weight gain in comparison to ≥70-year-old patients. The other age group categories which are 40–49, 50–59, and 60–69 have no difference regarding weight in comparison to ≥70-year-old patients. Gender and weight interval also have no significant difference on weight in binary regression model using the aforementioned variables.

## Discussion

Antipsychotics are used for a wide spectrum of mental illnesses. As they are used chronically, the side effects of these medications can cause an impact on the patients’ health. AIWG is considered one of the most prominent side effects of these drugs which can lead to the detrimental effects of metabolic syndrome. This study aims to measure the effects of antipsychotics with and without the concomitant use of metformin on body weight. The study outcomes revealed that patients in the metformin and antipsychotics group had less tendency to gain weight when compared to patients in the antipsychotics group.

In this study, we investigated the effect of metformin in reducing the AIWG. The statistical analysis reported that metformin concomitant use caused a significant reduction in the AIWG with a mean change of −0.04 kg as compared to a mean increase of 2.5 kg in patients in the antipsychotics group. These quantitative results are in line with the existing evidence that metformin is effective in controlling AIWG. A meta-analysis conducted by de Silva et al. (15) reported similar findings from several clinical studies where metformin effectively reduced the AIWG. Armen et al. reported that patients treated with metformin had a mean weight gain of 0.83 kg, while the placebo group had a mean weight gain of 2.2 kg (20). Regarding the qualitative results in our study, only 21.68% of patients in the antipsychotics group experienced weight loss as compared to 40.7% of patients who used antipsychotics and metformin. In addition, Wang et al. study reported that 7% of those using antipsychotics without metformin lost weight as compared to 40.7% of patients using metformin (22). In our study, only 38.37% of the patients in the metformin treated group experienced weight gain as compared to 64.4% of those in the antipsychotics group. Wu et al. concluded that 16.7% of patients in the metformin-treated group gained more than 7% of their body weight, as compared to 63.13% in the placebo group (23). These results are in the same line with this study outcome. However, some variability might be addressed in the previous randomized controlled trial by Wu et al., where it was reported that 16.7% in the metformin treated group gained weight compared to 38.37% in our study. We can attribute these differences to the fact that our study was a retrospective cohort study, which justifies, to some extent, the mild inconsistency with other studies.

Regarding the association between gender and weight, a study conducted by Lee et al. (24) suggested that female patients marginally have more weight gain than males. However, in this study, statistical analysis revealed no significant difference between gender and weight change. Importantly, the study outcomes exhibited that female patients appeared to take metformin with antipsychotics more than males even though there was no significant difference regarding weight and gender. The elevated proportion of females taking metformin with antipsychotics can be explained by some other indications for metformin use such as polycystic ovary syndrome as one of the obesity’s complications in females (16, 25).

The weight gain between different age groups is found to be elevated among the youngest group (20–29 years) and lowest among the group of 60–69 years old. This significance regarding AIWG in different age groups might be related to the use of Metformin since the concomitant use of metformin with antipsychotics was higher in the oldest (≥70 years) group as compared to the youngest age group (20–29 years). In similar line with this outcome, studies conducted by M. Dayabandara et al. and Lee S. Y. et al., reported that the amount of weight gain is highest in younger patients, then gradually decreased with the increase in age (5, 24). Little evidence supports our results on the increasing use of metformin among the oldest group of patients (≥70 years). However, we estimate that it could be due to comorbidities among elderly patients, namely type 2 diabetes. These results suggest paying more attention to weight gain among young patients.

Despite the current study outcomes, which suggest that there is no significant difference among various antipsychotics monotherapy, several reports revealed that antipsychotics, such as clozapine, olanzapine, and quetiapine can cause AIWG as side effects more than other antipsychotics (26–28). However, the mechanism by which these medications induce weight gain is not fully understood (29). On the other hand, there is also sufficient research that aripiprazole is one of the least weight gain inducing antipsychotics. Another study suggested that aripiprazole had significantly less impact on weight gain as compared to different antipsychotic drugs (12). Another research also demonstrated that aripiprazole may have an effect on its own reducing AIWG as an add-on treatment without the use of Metformin (5). These results support that there is a difference in weight between different antipsychotics, so if a patient with a psychiatric disease has obesity, a physician should consider antipsychotics with a minimum weight increase such as aripiprazole instead of medications that have a high impact on weight such as olanzapine or clozapine. However, as previously mentioned, this study concluded that there is no significant difference among different drugs which may be attributed to the presences of confounding factors, such as unequal distribution of Metformin usage or unequal distribution of patients’ numbers. In addition, statistical analysis revealed no significant difference between different weight intervals. In contrast to our outcomes, another study revealed that weight gain was increased in patients using antipsychotics for 6 months (3.5 kg), whereas in 3 months, the weight increased was only 2 kg from baseline (30).

By using a binary logistic regression model with the outcome of decrease/no change in weight and increase in weight, the independent variables of metformin usage, gender, age, and weight interval. Gender and weight interval outcomes are similar to the univariable analysis done in this study, which revealed that these two variables have no significant difference in weight. Also similar to the uni-variable analysis, usage of Metformin can significantly improve the weight since using antipsychotics alone is associated with increasing the weight gain by 2.35-folds. Also, in the binary logistic regression model, the highest age group who had weight gain are patients who are 20–29 years old. This is a dissimilarity of our univariable results which mention that the highest weight gain is in patients who are aged 20–29. These findings are clinically significant in guiding physicians to select the appropriate regimen for an acceptable duration, given that AIWG can be avoided or minimized using the previous results.

## Limitations and Future Directions

One of the limitations of this study is the retrospective design for measuring the desired outcomes. Prospective studies are more accurate in portraying the effect of antipsychotics due to privilege of selecting the participants and ability to reduce the confounding factors. Thus, future studies are guaranteed to prospectively measure the outcomes of antipsychotics and metformin concomitant use on AIWG. Even though this study excluded some conditions that cause weight fluctuations as mentioned in the methods section, many confounding factors such as patients with polypharmacy, lifestyle, physical activity, diet, and other comorbidities may cause weight changes were not measured. Also, some participants underwent some changes in their treatment regimen which in turn may have affected the duration of the medication used. To the best of our knowledge, the research in this field is not well-established in the Kingdom of Saudi Arabia; thus, additional prospective and multi-center studies should be implemented. Moreover, further studies are required to investigate the impact of AIWG on different age groups. Furthermore, additional research is necessary to demonstrate the potential role of other medications such as statins or incretin mimetics on reducing the AIWG (31).

## Conclusion

Statistically significant data displayed that antipsychotic drugs can cause weight gain among patients and if paired with metformin, it can reduce these effects or even cause weight loss. As weight gain has many side effects, it is important for psychiatrists to educate their patients about these side effects and suggest the appropriate modifications to their treatment regimen to prevent these effects.

## Data Availability Statement

The data analyzed in this study is subject to the following licenses/restrictions: the patients’ data are utilized for the research purpose, and the datasets are not publicly available since it contains personal information regarding the patients. Requests to access these datasets should be directed to AH, hakamia@ksau-hs.edu.sa.

## Ethics Statement

The studies involving human participants were reviewed and approved by the King Abdullah International Medical Research Center (KAIMRC) Institutional review board. Written informed consent for participation was not required for this study in accordance with the national legislation and the institutional requirements.

## Author Contributions

All authors listed have made a substantial, direct, and intellectual contribution to the work, and approved it for publication.

## Conflict of Interest

The authors declare that the research was conducted in the absence of any commercial or financial relationships that could be construed as a potential conflict of interest.

## Publisher’s Note

All claims expressed in this article are solely those of the authors and do not necessarily represent those of their affiliated organizations, or those of the publisher, the editors and the reviewers. Any product that may be evaluated in this article, or claim that may be made by its manufacturer, is not guaranteed or endorsed by the publisher.
